# High-Power Ultrasound (HPU) and Pulsed Electric Field (PEF) in the Hurdle Concept for the Preservation of Antioxidant Bioactive Compounds in Strawberry Juice—A Chemometric Evaluation—Part II

**DOI:** 10.3390/foods13040537

**Published:** 2024-02-09

**Authors:** Anica Bebek Markovinović, Višnja Stulić, Predrag Putnik, Nikša Bekavac, Branimir Pavlić, Sanja Milošević, Branko Velebit, Zoran Herceg, Danijela Bursać Kovačević

**Affiliations:** 1 Faculty of Food Technology and Biotechnology, University of Zagreb, Pierottijeva 6, 10000 Zagreb, Croatia; anica.bebek.markovinovic@pbf.unizg.hr (A.B.M.); vstulic@pbf.hr (V.S.); nbekavac@pbf.hr (N.B.); zherceg@pbf.hr (Z.H.); 2 Department of Food Technology, University North, Trg dr. Žarka Dolinara 1, 48000 Koprivnica, Croatia; 3 Faculty of Technology, University of Novi Sad, Blvd. Cara Lazara 1, 21000 Novi Sad, Serbia; bpavlic@uns.ac.rs (B.P.); sanjamilosevic9898@gmail.com (S.M.); 4 Institute of Meat Hygiene and Technology, Kaćanskog 13, 11040 Belgrade, Serbia; branko.velebit@inmes.rs

**Keywords:** hurdle concept, non-thermal technology, functional juice, bioactive compounds, storage

## Abstract

In this work, the influence of high-power ultrasound (HPU) followed by pulsed electric field (PEF) in the hurdle concept (HPU + PEF) on the content of biologically active compounds (BACs) and antioxidant activity in strawberry juices stored at 4 °C/7 days was investigated. The HPU was performed with an amplitude of 25% and pulse of 50% during 2.5, 5.0 and 7.5 min, while the PEF was performed with an electric field strength of 30 kV cm^−1^ and frequency of 100 Hz during 1.5, 3 and 4.5 min. The results obtained indicate that the synergy of the mechanisms of action for technologies in the hurdle concept plays a critical role in the stability of BACs and antioxidant activity. Juices treated with HPU + PEF hurdle technology and kept at 4 °C for 7 days showed a statistically significant decrease in all BACs, antioxidant capacity and pH. Shorter HPU + PEF treatment times favored the preservation of BACs in juices. Regarding total phenolic compounds, flavonols, condensed tannins and antioxidant capacity, optimization of hurdle parameters showed that a shorter HPU treatment time of 2.5 min provided the best yield of these compounds. In summary, by optimizing and adjusting the parameters of the HPU/PEF technology, it is possible to produce functional strawberry juice.

## 1. Introduction

A diet rich in fruits and vegetables has a helpful effect on human health and general well-being due to the effects of various ingredients, such as polyphenols, carotenoids, tocopherols, vitamins, minerals, fibers and others [1]. In recent years, fresh fruits and vegetables have been increasingly consumed in the form of pressed juices, smoothies and fermented beverages [2]. The quality of fruit juices depends on physical, organoleptic and microbiological aspects, as well as enzymatic activity. The shelf life of the product can be shortened by the action of enzymes, but also by the growth of microorganisms and/or oxidation reactions. In order to inactivate enzymes, but also to preserve health properties, fruit juices are traditionally preserved by heat treatment [3]. Nevertheless, increased temperatures have a negative impact, not only on the bioactive composition, but also on the color parameters and sensory properties, which is why there has been a growing interest in the application of non-thermal preservation methods in recent years [4].

Though non-thermal treatments do not appear to be as invasive as thermal treatments, the global effect depends on the matrix of the food to be processed [5]. Therefore, it is necessary to choose the most suitable non-thermal process with validated processing conditions to preserve all the nutrients and the original organoleptic properties [6]. Nowadays, consumers are increasingly attracted to minimally processed food without additives, proven safety and longer shelf life while maintaining biological and functional properties [7]. Due to these facts, the food industry combines different technologies in food processing, i.e., it uses the so-called concept of “hurdle technology”, in which a well-defined sequence of process hurdles is applied that the microorganisms present cannot “overcome” [8]. The aim of using “hurdle technology” is to simultaneously improve nutritional and sensory quality and increase food safety [9]. The main benefit of the use of hurdle processing, which consists of the combination of different technologies, is the synergistic effect of different mechanisms that achieve an improved preservation effect [9]. By carefully combining hurdles, each individual process can be carried out under much milder conditions than when applied separately. The resulting product can be considered microbiologically stable and at the same time exhibit improved functionality [8].

Recently, pulsed electric field (PEF) and high-power ultrasound (HPU) technologies have shown promising results in the processing of fruit juices to obtain stable and biologically valuable functional products [10,11,12]. PEF technology is characterized by the inactivation of microorganisms and enzymes, which can be a substitute to thermal pasteurization [13]. Depending on applied process parameters, such as the strength of the electric field or the number of pulses, the effect of PEF can produce different effects and can therefore be used for different purposes [14,15].

Although the mechanism of action at the cellular level is still not reliably defined, the most common explanation of the mechanism is supported by the phenomenon of electroporation and electropermeability of the cell membrane, whereby membrane damage occurs with the formation of (i)reversible ruptures as a result of exposure to an external electric field [16,17]. PEF processing can also provide products with improved antioxidant properties [18]. For example, strawberry juice treated with PEF shows a retention of antioxidant capacity of 75% to 100% in comparison to untreated juice [19]. The HPU is based on cavitation effect, which can lead to physical and chemical changes in the material. The effect of HPU creates a longitudinal mechanical wave in the liquid medium and an alternating pressure change occurs, i.e., phases of compression and expansion [17], which leads to the occurrence of cavitation. During cavitation, gas bubbles form in the medium, the volume of which increases from cycle to cycle until a critical size is reached. At the moment the critical size is reached, vapor condensation and implosion of the bubbles occur, and the molecules collide at high speed, creating the so-called shock waves. Shock waves cause very high temperatures (up to 5500 K) and pressures (up to 100 MPa). The formation of cavitation is in relation to processing parameters (e.g., frequency, intensity), the product characteristics (e.g., viscosity, density) and environmental conditions (e.g., temperature, pressure) [20].

The preservation of food and beverages using the hurdle concept is increasingly being researched to replace thermal preservation processes with sustainable technologies as thermal processing can have an undesirable effect on the food quality. In the last 5 years, only a few studies have studied the impact of hurdle technology on the quality of strawberry juice. The influence of the combination of ultrasound (40 kHz; 180 W; 0, 15 and 30 min) and natural antimicrobial additives (geraniol and pomegranate extract) on the microbial, sensory and nutritional quality of strawberry juice was studied by Tomadoni et al. [21]. Emamifar and Mohamadizadeh [22] studied the effect of ultrasound and antimicrobial ZnO nanostructure packaging on the quality of fresh strawberry juice (*Fragaria* × *anannasa* Duch., cultivar ‘Parous’) during cold storage for 35 days. However, the combination of HPU and PEF technologies has not yet been tested on strawberry juice, with the exception of a previously published paper, in which the technology was tested on strawberry juice in the PEF + HPU sequence [12]. Precisely because of the different mechanisms, it is of great importance to select the optimal sequence of hurdle technologies under optimized processing conditions. The aim of this work was therefore to investigate the effects of hurdle technology, i.e., the combination of HPU (amplitude 25%; pulse 50%; 2.5, 5.0 and 7.5 min) followed by PEF (30 kV cm^−1^; 100 Hz; 1.5, 3.0 and 4.5 min) on the stability of polyphenolic compounds and antioxidant capacity in strawberry juices during 7-day storage at 4 °C. In the final consideration, the results of this research will be compared with the results of previous research [12] to better understand the mechanism and influence of hurdle technology in the processing of functional strawberry juices.

## 2. Materials and Methods

### 2.1. Juice Samples

Strawberries (*Fragaria* × *ananassa* Duch, cultivar ‘Albion’) were grown and harvested in 2022 at Jagodar HB in Donja Lomnica, Croatia. After harvesting, the fruits were transported to the laboratory, de-stemmed, washed, dried and stored at −18 °C. The juice was processed in a Kuvings B6000 slow juicer (240 W, speed 60 rpm; VerVita d.o.o., Zagreb, Croatia). The experiment was carried out according to the experimental research design (Table 1).

### 2.2. High-Power Ultrasound (HPU) and Pulsed Electric Field (PEF) in Hurdle Concept for Processing Strawberry Juice

A total of 200 mL of strawberry juice was processed with HPU and then with PEF. For the HPU treatment, the Hielscher UP400St (400 W, 24 kHz) device (Hielscher Ultrasonics GmbH, Teltow, Germany) with a titanium horn Ø 22 mm was used, so that the maximum energy density was up to 300 W/cm^2^. The HPU process parameters were amplitude of 25% (11.5 μm), pulse of 50% and treatment times of 2.5, 5.0 and 7.5 min. The duty cycle between pause and ultrasound treatment was set to 50%, which means that an ultrasound treatment lasted 0.5 s with a pause of 0.5 s.

A batch PEF system with the HVG60/1 HIPEF device (Impel d.o.o., Zagreb, Croatia), consisting of a Magna control unit, a high-voltage power source and a high-voltage pulse generator, was used for the PEF treatment. The treatment chamber consisted of 2 circular plates assembled in the form of a cylinder with a capacity of 200 mL. The treatments were performed with an electric field strength of 30 kV cm^−1^ at a frequency of 100 Hz. The total treatment times were 1.5, 3.0 and 4.5 min with the pulses of 1 µs. The ground electrode and the high-voltage electrode were 2.5 cm apart. A total of 18 juices were treated with the hurdle concept, and two samples were control samples, which were untreated and served to compare the effect of the hurdle concept.

Throughout processing with the HPU, followed by PEF technology, the temperature was monitored before and after treatment using a PCE-777 infrared thermometer (PCE Instruments, Southampton, UK). The average initial temperature of the juices before processing was 15.67 °C, after the HPU treatment 19.55 °C and after the PEF treatment 17.97 °C; therefore, the effect of temperature can be ignored. At the end of each treatment, all the samples were kept in sterilized, hermetically sealed glass bottles. One batch of juices was analyzed immediately after treatment, and another batch was kept at 4 °C/7 days. All juice samples were tested for physico-chemical properties (pH and SSC), the content of BACs and antioxidant activity.

### 2.3. pH and Soluble Solids Content (SSC)

A Mettler Toledo FiveEasy pH meter (Mettler-Toledo GmbH, Greifensee, Switzerland) and digital refractometer (ATAGO Pal-3 digital refractometer, ATAGO Co., Tokyo, Japan) were used for determination of pH and SSC, respectively.

### 2.4. Extraction Procedure

An extraction protocol was adopted from the literature [23]. The strawberry juice sample (5 g) was mixed with 1% formic acid in 80% methanol (*v*/*v*) (20 mL). Ultrasound-assisted extraction was performed in an ultrasonic bath (DT 514 H Sonorex Digitec 13.5 L, 860 W, 40 kHz, Bandelin electronic, Berlin, Germany) at 50 °C for 15 min. After filtration, the supernatant was transferred to a volumetric flask (25 mL) and made up with the extraction solvent. The extracts were kept at −18 °C in an inert gas atmosphere until analysis.

### 2.5. The Content of Bioactive Compounds

For all spectrophotometric determinations, a spectrophotometer (LLG-uniSPEC 2 Spectrophotometer, Buch and Holm, Meckenheim, Germany) was used. Total phenolic content (TPC) was determined by the modified method described in the literature by measuring absorbance at 725 nm [24]. Hydroxycinnamic acids (HCAs) and flavonols (FLs) were determined by the spectrophotometric method using 1 g L^−1^ HCl solution in 96% ethanol and 2 g L^−1^ HCl solution in distilled water by measuring the absorbance at 320 nm and 360 nm, respectively [25]. In addition, a modified spectrophotometric method was used to determine condensed tannins (CTs) using a 25% solution of sulfuric acid in methanol and a 1% solution of vanillin in methanol by measuring the absorbance at 500 nm [26]. The results of TPC were expressed as mg gallic acid equivalent (GAE) per 100 g sample, and HCA content and FLs were expressed as mg chlorogenic acid equivalent (CAE) per 100 g sample and mg quercetin equivalent (QE) per 100 g sample, respectively. The results of CTs were expressed as mg catechin equivalent (CA) per 100 g of sample. The determination procedure is described in detail in the first part of the published research paper [12].

### 2.6. In Vitro Antioxidant Activity Assay

In this study, two assays DPPH and FRAP, based with specific mechanisms of action, were used [27,28]. For the DPPH assay, a 0.5 mM DPPH solution was used, and the colorimetric response was recorded at 517 nm. For the FRAP assay, the FRAP reagent was used, the reaction was thermostatted at 37 °C for 10 min and the absorbance was recorded at 593 nm. The results obtained with both assays were expressed as µmol Trolox equivalent (TE) per 100 g of sample. The detailed protocol can be found in the first part of the published research paper [12].

### 2.7. Statistical Analysis

Full factorial randomized experimental designs were used for the experiments (n = 40) (Table 1). All BAC contents and antioxidant activities were the dependent variables. HPU treatment (2.5, 5.0 and 7.5 min), PEF treatment (1.5, 3.0 and 4.5 min) and storing time (0 and 7 days) were all independent factors. For the experimental dataset, descriptive statistics were used to evaluate the basic data. Multiple analysis of variance (MANOVA) was used to examine differences between treatments (continuous variables). To evaluate correlations between pairs of continuous variables, the Pearson coefficient was used. Exploratory hierarchical Ward cluster analysis was used to measure standardized similarities in samples, and the Kruskal–Wallis test was used for nonparametric analysis. Significance levels for rejection of a null hypothesis were α ≤ 0.05 for all tests. IBM SPSS Statistics (v.24) was used for analyses, and Statgraphics Centurion^®^ (StatPoint Technologies, Inc., Warrenton, VA, USA) was used for experimental design.

## 3. Results and Discussion

### 3.1. Chemometrics Evaluation of Hurdle-Treated Samples against Untreated Samples

**Figure 1:**
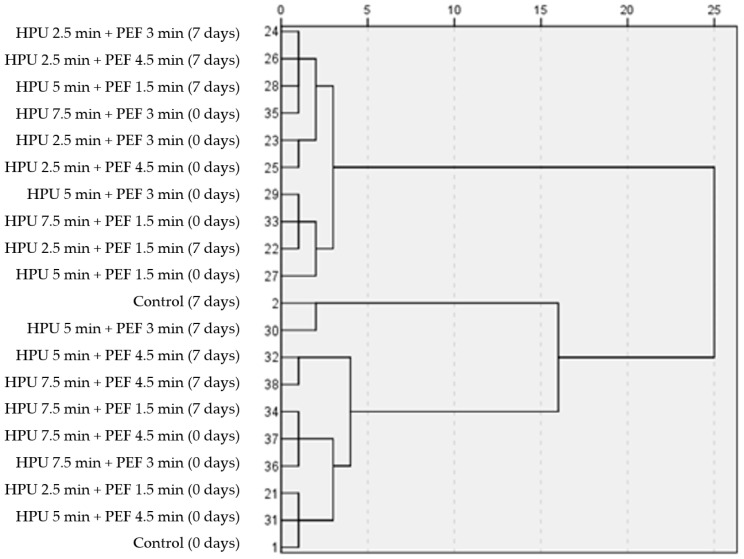
Results of the hierarchical clustering for averaged and standardized juice samples.

An exploratory hierarchical Ward’s cluster analysis was performed to determine the influence of hurdle processing (HPU + PEF) on the quality of strawberry juice. When all samples were analyzed for standardized similarities (TPC, HCA, FL, CT, DPPH, FRAP, SSC, pH), the samples treated with HPU for 2.5 min and 1.5 min with PEF followed by HPU for 5.0 min and 4.5 min with PEF were most similar to the control samples at 0 days of storage. These findings were similar to our previous results where we studied the reverse order of hurdle technologies. On day 0 of storage, samples treated with PEF for 1.5 min and 2.5 min with HPU were most similar to controls [12]. On day 7 of storage, samples treated with HPU for 5.0 min and 3.0 min with PEF were most similar to controls (Figure 1).

However, compared to the PEF + HPU combination treatment, the samples most similar to the untreated sample after one week of storage differ in the duration of the PEF treatment (PEF + HPU—1.5 min and HPU + PEF—3 min).

**Figure 2:**
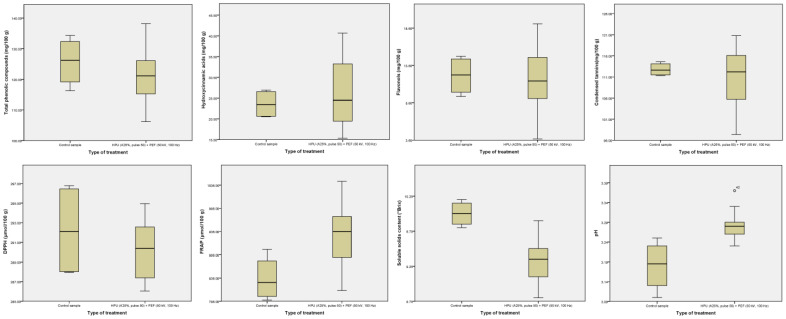
Median values of BAC content, antioxidant activity, SSC and pH in control vs. hurdle-treated samples.

The untreated samples did not differ from the samples treated with the hurdle technology with respect to the BACs analyzed and antioxidant activity. There were only differences in FRAP and pH, where the experimental samples had higher median values than the control samples, and in SSC, when this was reversed (i.e., the control samples were more acidic than the hurdle samples, had lower FRAP antioxidant activity and higher SSC; Figure 2). The differences in the antioxidant capacity of DPPH and FRAP in the treated juice samples may be attributed to the different sensitivity of these methods to the same fruit BACs [29]. In addition, the treated strawberry juice samples exhibited decreased SSC levels, which is consistent with the results of Koners et al. [30], who found a reduction in SSC levels in the excess sludge during the PEF treatment parameters (energy input of 100 kJ kg^−1^ at 15 kV cm^−1^ and sludge retention time of 14 days).

**Figure 3:**
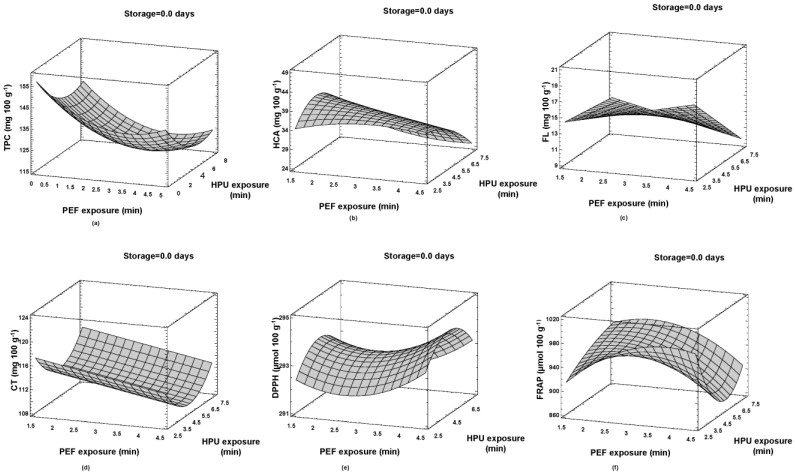
Optimal processing parameters (HPU + PEF) for highest level of BACs and antioxidant activity: (**a**) total phenolic content (TPC mg 100 g^−1^); (**b**) hydroxycinnamic acids (HCAs mg 100 g^−1^); (**c**) flavonols (FLs mg 100 g^−1^); (**d**) condensed tannins (CTs mg 100 g^−1^); (**e**) DPPH assay (DPPH µmol 100 g^−1^); and (**f**) FRAP assay (FRAP µmol 100 g^−1^).

Compared to the results of the reverse treatment sequence (PEF + HPU) [12], there are still common results of an increase in pH for the treated samples, which could be attributed to the possibility of electrolysis or electrochemical interactions during the treatment [31]. Table 2 shows the numerical values of the Kruskal–Wallis test of the hurdle-treated samples compared to the untreated samples, which correlates with the graphical representation in Figure 3.

### 3.2. The Changes in Strawberry Juices after Hurdle Processing and Storage with Respect to BACs, Antioxidant Activity, SSC and pH

Table 3 shows the effects of the processing parameters of the hurdle technology (HPU + PEF) and storage on the content of BACs, antioxidant activity, SSC and pH of the treated strawberry juices. In the previous study, the effects of the hurdle concept, but in the order of PEF + HPU, on the stability of BACs, SSC and pH during storage were investigated [12]. This study therefore aimed to investigate the effect of different sequences of these technologies on the quality of functional strawberry juices and to explore the synergy of these technologies in maintaining quality.

The average values of BACs in the treated juice samples were as follows: 120.56 ± 0.44 mg 100 g^−1^ TPC, 27.01 ± 0.25 mg 100 g^−1^ HCA, 11.55 ± 0.16 mg 100 g^−1^ FL and 109.99 ± 0.31 mg 100 g^−1^ CT. Yildiz et al. [32] found a slightly higher TPC (137.59 ± 1.93 mg GEA 100 mL^−1^) in strawberry juice after the HPU treatment, as well as after the PEF treatment (144.97 ± 1.52 mg GEA 100 mL^−1^), but the authors treated the clear strawberry juice under different operating conditions for the PEF and HPU technologies. Comparing the results obtained with the previous [12], it can be seen that the average values of TPC, HCA and CT were higher in the samples treated with HPU + PEF than in the samples treated with PEF + HPU. The average value of antioxidant activity measured by the DPPH method for the juice samples treated first with PEF and then with HPU technology was 293.93 ± 0.09 μmol 100 g^−1^, while it was 290.24 ± 0.11 μmol 100 g^−1^ for the juices treated in the reverse order. The opposite trend was observed for the FRAP method, where an average value of 852.41 ± 5.42 μmol 100 g^−1^ was obtained for juice samples treated first with the PEF and then with the HPU technology, and an average value of 926.18 ± 5.51 μmol 100 g^−1^ was obtained for juices treated in reverse order of technology. Regarding the antioxidant activity, the results of the same samples measured by different methods for the determination of antioxidant activity sometimes differ due to the different sensitivity of these methods for the same BAC structures [29]. Also, the antioxidant activity measured by DPPH and FRAP showed lower values when treated with HPU + PEF compared to PEF + HPU. These trends in the results could be based on the assumption that electroporation is less damaging to cell membranes than cavitation during HPU treatment, which is more damaging to membranes [33]. These results suggest that there is a synergistic relationship between the mechanisms of action of these two technologies in the processing of strawberry juices and that the order of these technologies in the hurdle concept influences the stability of BACs and antioxidant activity.

All juice samples showed higher TPC, HCA, FL, CT, DPPH, FRAP and SSC on the first day of storage compared to day 7. However, the pH remained constant for all samples throughout the storage period. After 7 days of storage, there was a significant decrease in TPC (6.2%) and CT (4.56%) with the greatest decrease observed in HCA (36.32%) and FL (33.26%). SSC also decreased during the storage period. However, when the hurdle concept included PEF + HPU, higher proportions of all BACs were determined on the first day of storage, with the exception of CT, where higher concentrations were observed after 7 days of storage [12]. PEF treatment, applied in several studies, also promoted the extraction of tannins, and PEF showed significant effects on the condensation of tannins [34,35]. Thus, these results suggest that the sequence of technologies applied may have an impact on the stability of CTs during storage.

The antioxidant activity of juices treated with hurdles (DPPH and FRAP) decreased during storage in juice samples treated with both hurdle concepts (HPU + PEF, PEF + HPU) [12]. The observed decrease could be related to an enhanced tendency for polymerization of the polyphenols, which reduces the availability of hydroxyl groups for antioxidant potential. A higher degree of polymerization leads to improved molecular complexity and stericity, which may result in a lower availability of hydroxyl groups to scavenge DPPH radicals, leading to a corresponding decrease in antioxidant capacity [36]. In the study by Odriozola-Serrano et al. [37], the antioxidant capacity measured with the DPPH and ABTS methods after storage (7 days/4 °C) was higher in treated juices than in fresh strawberry juice. From this, the authors conclude that processing plays an imperative role in obtaining safe and stable juices, but also supports to preserve their antioxidant potential during storage [37].

In order to better understand the synergetic relationships between the two technologies, the influence of both technologies on the quality of the juices tested was investigated. The process parameters were previously optimized for both HPU and PEF technologies [11], so the influence of treatment duration was investigated in this study.

A longer duration of HPU treatment resulted in a statistically significant decrease in the values of TPC, FL, CT and FRAP. As already mentioned for storage, the decrease was greatest for FL at 33%. HCA, DPPH and SSC increased with a 5 min treatment and decreased significantly thereafter. One possible reason for the decrease in the content of BACs is their degradation upon prolonged exposure to HPU, which could be due to the oxidative degradation of phenolic compounds. In addition, the free radicals generated by acoustic cavitation can potentially oxidize polyphenolic compounds or induce the hydrolysis of the phenolic glycoside forms and promote the formation of aglycone structures that are less stable than their glycosidic moieties [38]. The pH remained constant without influence of HPU duration. However, this trend was not observed in the PEF + HPU hurdle treatment. Here, it was shown that the HPU treatment of 5 min gave the best results for almost all quality parameters investigated [12].

Prolonged PEF treatment led to a significant decrease in TPC, HCA, FL and CT. Interestingly, the antioxidant activity measured with DPPH was highest at the longest PEF duration (5 min) while no significant effect of PEF duration was observed for the antioxidant activity measured with the FRAP method. The results obtained contradict the research results of Odriozola-Serrano et al. [37], in which the authors observed a significant decrease in the antioxidant capacity of strawberry juice with an increasing duration of PEF treatment (100–2000 µs) and electric field strength (20–35 kV cm^−1^). One possible explanation is that as a result of PEF treatment, various reactions such as hydroxylation, methylation, isoprenylation, dimerization and/or glycosylation, which induce modifications between the different phenolic compounds, may occur to different extents during processing, so that such new structures may affect the antioxidant capacity [39]. Moreover, the duration of PEF treatment had no significant effect on the application of pH, but SSC decreased significantly with a longer treatment duration. This trend in the stability of BACs was not observed with the reverse order of processing technologies (PEF + HPU) [12], suggesting that the same technologies have a different synergistic effect on the stability of BACs depending on whether they are applied as the first or second technology in the hurdle sequence.

The HPU treatment of 2.5 min in combination with PEF (1.5–4.5 min) had no statistically significant effect on the values of BACs, DPPH and pH. The antioxidant activity measured with the FRAP assay was higher when the hurdle treatment lasted longer (PEF at 3 min and 4.5 min). The pH of the treated juices was constant, while the SSC was highest in the shortest treatment (HPU 2.5 min + PEF 1.5 min). In a study where PEF with the shortest treatment time (1.5 min) was combined with HPU treatments (2.5–7.5 min), there were still significant changes in the concentrations of FL and CT, and DPPH, again confirming that the sequence of PEF and HPU technologies plays an important role in the stability of BACs in strawberry juices [12].

The impact of ultrasound and PEF on the quality of spinach juice was investigated by Faisal Manzoor et al. [40]. First, an ultrasonic treatment (40 kHz, 200 W, 30 °C, 21 min) was performed in an ultrasonic bath, followed by a PEF treatment (1 kHz, 60 mL min^−1^, 30 °C, 335 µs, 9 kV cm^−1^). As in previous studies, higher levels of TPC (12%), flavonoids (10%), FL (23%), antioxidant capacity, anthocyanins (15%), carotenoids (18%), chlorophyll (17%) and vitamins were found compared to untreated samples and samples treated individually with PEF and HPU. The authors also note that the membrane permeabilization induced by PEF treatment enhances the extraction of intracellular contents, leading to an increase in extraction effectiveness and yield of intracellular metabolites. In addition, the authors note that the increase in TPC during sonication is likely related to the release of bound phenols due to cavitation-induced damage to the cell membrane.

However, the extension of HPU treatment to 5 min in combination with PEF (1.5–4.5 min) did not change the contents of FL, DPPH and pH, but led to a decrease in the contents of TPC, HCA, CT and FRAP. Interestingly, the SSC increased after 3 min of PEF exposure and decreased with further exposure, but not below the initial level. Comparing the previous results, where the HPU treatment lasted 2.5 min, with these, where the HPU treatment time is twice as long (5 min), it can be seen that the prolongation of the HPU treatment still has a significant impact on the structures of certain BACs and thus strongly influences their stability. This indicates that both technologies need to be carefully optimized to produce high-quality functional strawberry juices.

A further extension of the HPU application time to 7.5 min followed by treatment with PEF lowers the content of all BACs, while the antioxidant activity remains unchanged. Here, all bioactive compounds showed the highest levels at the PEF treatment (1.5 min), but this trend changed at a PEF treatment of 3 min. PEF treatment at 3 min together with HPU at 7.5 min had a negative effect on the contents of TPC and HCA. The content of CT decreased with PEF treatment at 4.5 min. Interestingly, the most invasive treatment for FL stability was HPU 7.5 min + PEF 4.5 min.

Furthermore, FRAP, DPPH and pH were not affected by this combination of hurdle technologies (HPU 7.5 min + PEF 1.5–4.5 min), while SSC decreased with increasing duration. Here in the HPU + PEF combination, without influence of treatment duration, no statistical changes in DPPH values were observed, although variations in BACs were detected. This could be due to the different contributions to antioxidant capacity by different chemical bioactive structures [41], as not all BACs in strawberry juice were determined in this study. However, in the reverse sequence treatment, PEF + HPU, longer treatments favored higher DPPH values, while in the longest treatments (PEF 4.5 min + HPU 2.5/5/7.5 min), there was no significant difference depending on the selected time combinations. In the PEF + HPU sequence, FRAP values were also unchanged in the PEF 1.5 min + HPU 2.5/5/7.5 min and PEF 3.0 min + HPU 2.5/5/7.5 min treatments, and the difference only occurred in the longest PEF treatment of 4.5 min [12]. These results confirm once again that the sequence of combinations as well as the optimization of PEF and HPU technologies significantly influence each tested parameter, supporting the thesis that their synergistic effect is of crucial importance for the final quality of processed strawberry juices.

To date, no data on ultrasound (US) hurdle treatment and PEF technology in the processing of strawberry juices have been found in the literature with which to compare the results obtained.

In a recent study, the hurdle concept was applied to the processing of strawberry juice by combining atmospheric cold plasma (dielectric barrier discharge at 60 kV, 50 Hz, 10 and 15 min) and hydrothermal treatment (121 °C, 10 min at 10 lbf in^−2^) to preserve the antioxidant bioactives. The authors concluded that a 10 min cold plasma treatment combined with hydrothermal processing preserves the biological quality of the treated strawberry juices [42]. Manzoor et al. [43] investigated the effects of the combination of US (40 kHz, 200 W, 35 °C, 20 min), PEF (flow rate 40 mL min^−1^, 18 kV cm^−1^, 500 µs, 1 kHz) and their combination PEF + US on the BAC content of almond extract. According to their results, the TPC increased in samples treated separately with PEF and US and in the samples treated with a combination of PEF + US compared to untreated samples. This study shows that the combination of PEF + US leads to higher yields of all analyzed BACs, such as TPC, TF, CT and total anthocyanins, than when these two technologies were used independently. The authors conclude that the reason for the higher content in the PEF + US treatment could be due to the different chemical effects of PEF and US on the plant matrix.

### 3.3. Optimization of Hurdle Technology Operating Parameters for the Treatment of Strawberry Juice

As shown in Table 4, all polyphenolic groups and DPPH decreased significantly during prolonged storage of strawberry juice after treatment with HPU and PEF, while no correlation was found for pH and SSC. These results differ from those obtained by PEF + HPU treatment [12], where storage has no significant effect on TPC and FL and a positive effect on the increase in CT. However, these results are in accordance with those of Nadeem et al. [44], who discovered that mixed carrot and grape juices treated with ultrasound (20 kHz; amplitude 70%; 2, 4 and 6 min) showed a gradual decrease in phenolics and flavonoids content with prolonged storage, but less than untreated or chemically preserved juice. Initial exposure to HPU strongly decreased TPC, FL, CT and FRAP values while the other variables did not correlate. Also in this case, the results differ from previously reported work [12], where HPU treatment had no effect on BAC content.

As expected, TPC was strongly positively correlated with all polyphenolic groups and with the two corresponding antioxidant activities (DPPH and FRAP), but not with SSC and pH. In other words, with the increase in all polyphenolic groups, the TPC and their antioxidant activity increased, which is quite logical and perhaps indicates a good quality of analytical measurement of polyphenols. These results are partly different from the previously mentioned results [12], where TPC correlated positively only with CT and FRAP values.

HCA was strongly positively correlated with FL, CT, DPPH and SSC, indicating the expected correlation between the measured variables (e.g., relationship of HCA to condensation to tannins). FL also showed a significant positive correlation with CT content and both antioxidant activities. These results are in partial accordance with those obtained by PEF + HPU treatment [12], where HCAs were significantly correlated with all BAC, DPPH and pH values, while FLs were significantly positively correlated only with DPPH. CT was correlated significantly positively with the values of both antioxidant capacities (Table 4). These results are in partial agreement with earlier studies with PEF + HPU treatment [12], in which CT is significantly negatively correlated with DPPH and positively correlated with FRAP and pH values. As for antioxidant capacity, DPPH was strongly positively correlated with HCA, FL and CT, implying that they are mainly responsible for the antioxidant activity in the samples. FRAP correlated mainly with FL and CT (and the previously mentioned TPC), suggesting that they are the main contributors to the antioxidant activity measured by this assay. The SSC value correlates significantly positively with pH, which was not the case in the previous studies with PEF + HPU treatment, where no correlation was found [12].

The optimization of the HPU + PEF process parameters was carried out to find the operating parameters with which the highest content of BACs and antioxidant activity can be achieved (Table 5). For all BACs and antioxidant activity, the highest levels were obtained with a shorter storage time (0 days). The same results for storage time were obtained for the optimal treatment of PEF + HPU in a previously published work [12]. The highest content of TPC/CT of 129.35 and 117.19 mg 100 g^−^^1^ was obtained for a 2.5 min HPU treatment and a 1.5 min PEF treatment, respectively. The highest content of HCA required a longer HPU treatment of 4.7 min, but a similar PEF treatment (as for TPC/CT) of 1.5 min for the highest content of 39.15 mg 100 g^−^^1^. The highest content of FL (18.04 mg 100 g^−^^1^) in the samples was observed with an HPU treatment of 2.5 min and a PEF treatment of 4.5 min. Overall, the best DPPH activity (294.25 µmol 100 g^−^^1^) was observed at 5 min HPU and 4.5 min PEF. Finally, the strongest antioxidant activity, assessed by FRAP (987.75 µmol 100 g^−^^1^), was observed at 2.5 min HPU exposure and 3.8 min PEF. In summary, higher levels of polyphenols and antioxidant activity favored shorter exposure times, both for HPU (up to 2.5 min) and PEF (up to 1.5 min). The shorter exposure time for certain technologies could be explained by the effective and rapid destabilization of the cell membrane under the influence of electroporation and cavitation phenomena, consequently, by the easier extraction of said compounds into the extracellular space [45,46,47].

On the one hand, when comparing PEF + HPU and HPU + PEF in terms of optimal BAC quantity and antioxidant activity [12], it can be seen that the HPU + PEF hurdle concept was more favorable as it can produce strawberry juices with improved functional properties (Figure 3). On the other hand, comparing the results of PEF + HPU and HPU + PEF [12] in terms of required operation time, it can be seen that when PEF is the first technology in the hurdle concept, followed by HPU, the longer HPU treatment is required to obtain the highest content of BACs studied, with the exception of TPC, and thus a higher energy input is required to obtain higher yields of the targeted BACs. Even without influencing which technology is the first hurdle in processing, the same PEF duration is required for PEF + HPU and HPU + PEF to obtain the maximum content of BACs. However, the strawberry juices treated with PEF + HPU achieved maximum antioxidant activity with a shorter PEF duration compared with the HPU + PEF combination, which again speaks in favor of the economically more favorable PEF + HPU processing.

## 4. Conclusions

Strawberry juice obtained from the ‘Albion’ cultivar can be considered as a functional food because of its high level of BACs and noteworthy antioxidant activity. Hurdle technology could be a good choice to prevent the quality of treated juices. The selection of HPU and PEF technologies in the hurdle concept proved to be a good resolution in the processing of functional strawberry juices. The results obtained show that it is not only important to optimize the process parameters of each technology, but also that the order of technology application must be studied, as it significantly affects the bioactive potential and antioxidant capacity of the treated strawberry juices. This supports the thesis that the synergistic effect of the selected technologies is crucial when defining the hurdle concept and should not be neglected.

The results obtained show that the sequence of HPU + PEF technologies was more efficient in the preservation of BACs in strawberry juices than PEF + HPU treatments since greater stability of the studied compounds was achieved with the same treatment durations. Considering the order of the applied technologies, it can be seen that the stability of TPC, CTs and pH is better influenced by the order of HPU + PEF treatment than by PEF + HPU. The results related to antioxidant activity obtained by the FRAP method show the same trend.

When the samples were initially treated by HPU, a longer duration of HPU treatment decreased the content of all BACs. The same was observed with PEF treatment. As with the combination of PEF + HPU treatment, shorter HPU + PEF treatments had a more favorable effect on the retention of BACs in strawberry juice. After the HPU + PEF treatment, reduced contents of all BACs during storage were observed. The results of the analysis of the effects of storage on the antioxidant capacity of the samples indicate a decrease in the antioxidant capacity, regardless of the order of treatment applied.

In terms of TPC, FL and CT as well as FRAP antioxidant capacity, the shorter HPU treatment time (2.5 min) is most favorable when the hurdle technology is applied in the HPU + PEF combination; while, for HCA and DPPH, the antioxidant capacity requires a slightly longer treatment. As with the combination of PEF + HPU, higher levels of BACs are obtained with a shorter PEF treatment (less than 5 min).

In summary, hurdle technology as an innovative processing concept with selected PEF and HPU technologies can be considered as a sustainable technology that has a broad industrial application perspective in the processing of functional strawberry juices.

## Figures and Tables

**Table 1:** Experimental research design.

Juice Sample	Storage (Days)	Hurdle Processing	HPU Treatment (min)	PEF Treatment (min)
1	0	Control	0	0
2	0	HPU + PEF	2.5	1.5
3	0	HPU + PEF	2.5	3.0
4	0	HPU + PEF	2.5	4.5
5	0	HPU + PEF	5.0	1.5
6	0	HPU + PEF	5.0	3.0
7	0	HPU + PEF	5.0	4.5
8	0	HPU + PEF	7.5	1.5
9	0	HPU + PEF	7.5	3.0
10	0	HPU + PEF	7.5	4.5
11	7	Control	0	0
12	7	HPU + PEF	2.5	1.5
13	7	HPU + PEF	2.5	3.0
14	7	HPU + PEF	2.5	4.5
15	7	HPU + PEF	5.0	1.5
16	7	HPU + PEF	5.0	3.0
17	7	HPU + PEF	5.0	4.5
18	7	HPU + PEF	7.5	1.5
19	7	HPU + PEF	7.5	3.0
20	7	HPU + PEF	7.5	4.5

Control—untreated strawberry juice; and HPU + PEF—strawberry juice samples processed with HPU and PEF technology in hurdle concept.

**Table 2:** Results for Kruskal–Wallis test (hurdle treatment vs. control samples).

	TPC	HCA	FL	CT	DPPH	FRAP	SSC	pH
Chi-Square	1.37	0.10	0.03	0.03	1.17	6.84	8.9	9.11
df	1	1	1	1	1	1	1	1
Sig.	0.24	0.75	0.86	0.86	0.28	≤0.01 *	≤0.01 *	≤0.01 *

* Kruskal–Wallis test is significant at *p* ≤ 0.05. TPC—total phenolic content (mg 100 g^−1^); HCA—hydroxycinnamic acid (mg 100 g^−1^); FL—flavonol (mg 100 g^−1^); CT—condensed tannin (mg 100 g^−1^); antioxidant activity—DPPH (µmol 100 g^−1^) and FRAP (µmol 100 g^−1^); and SSC—soluble solids content (°Brix).

**Table 3:** The influence of hurdle technology (HPU + PEF) on BACs, antioxidant activity, SSC and pH of strawberry juices throughout storage.

32	n	TPC	HCA	FL	CT	DPPH	FRAP	SSC	pH
Storage		*p* ≤ 0.01 ^†^	*p* ≤ 0.01 ^†^	*p* ≤ 0.01 ^†^	*p* ≤ 0.01 ^†^	*p* ≤ 0.01 ^†^	*p* = 0.03 ^†^	*p* ≤ 0.01 ^†^	*p* = 0.08 ^‡^
0 days	18	124.35 ± 0.63 ^a^	33.01 ± 0.67 ^a^	13.86 ± 0.23 ^a^	113.58 ± 0.45 ^a^	292.86 ± 0.15 ^a^	940.18 ± 7.79 ^a^	9.36 ± 0.01 ^a^	3.29 ± 0.01 ^a^
7 days	18	116.78 ± 0.63 ^b^	21.02 ± 0.67 ^b^	9.25 ± 0.23 ^b^	108.40 ± 0.45 ^b^	287.62 ± 0.15 ^b^	913.44 ± 7.79 ^b^	9.30 ± 0.01 ^b^	3.27 ± 0.01 ^a^
HPU		*p* ≤ 0.01 ^†^	*p* ≤ 0.01 ^†^	*p* ≤ 0.01 ^†^	*p* ≤ 0.01 ^†^	*p* ≤ 0.01 ^†^	*p* ≤ 0.01 ^†^	*p* ≤ 0.01 ^†^	*p* = 0.20 ^‡^
2.5 min	12	126.02 ± 0.77 ^a^	28.35 ± 0.82 ^a^	13.83 ± 0.28 ^a^	116.30 ± 0.55 ^a^	289.81 ± 0.19 ^b^	961.62 ± 9.54 ^a^	9.27 ± 0.01 ^b^	3.27 ± 0.01 ^a^
5.0 min	12	118.52 ± 0.77 ^b^	29.93 ± 0.82 ^b^	11.71 ± 0.28 ^b^	108.06 ± 0.55 ^b^	290.96 ± 0.19 ^a^	905.76 ± 9.54 ^b^	9.63 ± 0.01 ^a^	3.29 ± 0.01 ^a^
7.5 min	12	117.14 ± 0.77 ^b^	22.76 ± 0.82 ^c^	9.14 ± 0.28 ^c^	108.61 ± 0.55 ^b^	289.95 ± 0.19 ^b^	913.06 ± 9.54 ^b^	9.08 ± 0.01 ^c^	3.28 ± 0.01 ^a^
PEF		*p* ≤ 0.01 ^†^	*p* ≤ 0.01 ^†^	*p* ≤ 0.01 ^†^	*p* ≤ 0.01 ^†^	*p* ≤ 0.01 ^†^	*p* = 0.28 ^‡^	*p* ≤ 0.01 ^†^	*p* = 0.16 ^‡^
1.5 min	12	124.48 ± 0.77 ^a^	28.66 ± 0.82 ^a^	12.64 ± 0.28 ^a^	113.12 ± 0.55 ^a^	290.12 ± 0.19 ^b^	931.60 ± 9.54 ^a^	9.41 ± 0.01 ^a^	3.29 ± 0.01 ^a^
3.0 min	12	118.07 ± 0.77 ^b^	27.31 ± 0.82 ^b^	10.96 ± 0.28 ^b^	110.70 ± 0.55 ^b^	289.70 ± 0.19 ^b^	934.81 ± 9.54 ^a^	9.38 ± 0.01 ^a^	3.27 ± 0.01 ^a^
4.5 min	12	119.14 ± 0.77 ^b^	25.06 ± 0.82 ^c^	11.07 ± 0.28 ^b^	109.14 ± 0.55 ^b^	290.91 ± 0.19 ^a^	914.03 ± 9.54 ^a^	9.20 ± 0.01 ^b^	3.27 ± 0.01 ^a^
HPU + PEF (hurdle)		*p* = 0.10 ^‡^	*p* = 0.10 ^‡^	*p* = 0.20 ^‡^	*p* = 0.07 ^‡^	*p* = 0.82 ^‡^	*p* = 0.05 ^†^	*p* ≤ 0.01 ^†^	*p* = 0.06 ^‡^
2.5 min + 1.5 min	12	129.54 ± 2.04 ^a^	28.43 ± 1.42 ^a^	13.47 ± 1.55 ^a^	115.87 ± 2.09 ^a^	289.55 ± 0.32 ^a^	918.05 ± 16.33 ^b^	9.43 ± 0.02 ^a^	3.30 ± 0.01 ^a^
2.5 min + 3.0 min	12	123.06 ± 2.04 ^a^	29.77 ± 1.42 ^a^	14.70 ± 1.55 ^a^	118.87 ± 2.09 ^a^	289.79 ± 0.32 ^a^	981.50 ± 16.33 ^a^	9.25 ± 0.02 ^b^	3.25 ± 0.01 ^a^
2.5 min + 4.5 min	12	125.46 ± 2.04 ^a^	26.85 ± 1.42 ^a^	13.30 ± 1.55 ^a^	114.15 ± 2.09 ^a^	290.08 ± 0.32 ^a^	985.32 ± 16.33 ^a^	9.13 ± 0.02 ^c^	3.26 ± 0.01 ^a^
HPU + PEF (hurdle)		*p* ≤ 0.01 ^†^	*p* ≤ 0.01 ^†^	*p* = 0.08 ^‡^	*p* ≤ 0.01 ^†^	*p* = 0.82 ^‡^	*p* = 0.02 ^†^	*p* ≤ 0.01 ^†^	*p* = 0.06 ^‡^
5.0 min + 1.5 min	12	123.21 ± 0.65 ^a^	32.73 ± 0.83 ^a^	12.94 ± 1.55 ^a^	113.01 ± 0.91 ^a^	290.87 ± 0.32 ^a^	951.30 ± 13.70 ^a^	9.33 ± 0.02 ^a^	3.28 ± 0.01 ^a^
5.0 min + 3.0 min	12	113.64 ± 0.65 ^b^	30.59 ± 0.83 ^a^	10.92 ± 1.55 ^a^	103.26 ± 0.91 ^b^	289.88 ± 0.32 ^a^	883.21 ± 13.70 ^b^	9.90 ± 0.02 ^b^	3.29 ± 0.01 ^a^
5.0 min + 4.5 min	12	118.70 ± 0.65 ^c^	26.46 ± 0.83 ^b^	11.27 ± 1.55 ^a^	107.90 ± 0.91 ^c^	292.14 ± 0.32 ^a^	882.76 ± 13.70 ^b^	9.68 ± 0.02 ^c^	3.30 ± 0.01 ^a^
HPU + PEF (hurdle)		*p* = 0.02 ^†^	*p* ≤ 0.01 ^†^	*p* ≤ 0.01 ^†^	*p* ≤ 0.01 ^†^	*p* = 0.82 ^‡^	*p* = 0.10 ^‡^	*p* ≤ 0.01 ^†^	*p* = 0.06 ^‡^
7.5 min + 1.5 min	12	120.68 ± 1.33 ^a^	24.83 ± 0.57 ^a^	11.52 ± 0.37 ^a^	110.49 ± 0.70 ^a^	289.93 ± 0.32 ^a^	925.44 ± 19.10 ^a^	9.48 ± 0.02 ^a^	3.29 ± 0.01 ^a^
7.5 min + 3.0 min	12	117.50 ± 1.33 ^a,b^	21.57 ± 0.57 ^b^	7.27 ± 0.37 ^c^	109.97 ± 0.70 ^a^	289.42 ± 0.32 ^a^	939.74 ± 19.10 ^a^	8.98 ± 0.02 ^b^	3.28 ± 0.01 ^a^
7.5 min + 4.5 min	12	113.25 ± 1.33 ^b^	21.86 ± 0.57 ^b^	8.63 ± 0.37 ^b^	105.38 ± 0.70 ^b^	290.50 ± 0.32 ^a^	874.00 ± 19.10 ^a^	8.80 ± 0.02 ^c^	3.26 ± 0.01 ^a^
Dataset average	36	120.56 ± 0.44	27.01 ± 0.25	11.55 ± 0.16	109.99 ± 0.31	290.24 ± 0.11	926.18 ± 5.51	9.33 ± 0.01	3.28 ± 0.01

Results are expressed as mean ± standard error. Values represented with different letters are statistically different at *p* ≤ 0.05; ^†^ significant factor in multifactor analysis; and ^‡^ not significant factor in multifactor analysis. TPC—total phenolic content (mg 100 g^−1^); HCA—hydroxycinnamic acid (mg 100 g^−1^); FL—flavonol (mg 100 g^−1^); CT—condensed tannin (mg 100 g^−1^); antioxidant activity—DPPH (µmol 100 g^−1^) and FRAP (µmol 100 g^−1^); and SSC—soluble solids content (°Brix). HPU—high-power ultrasound (amplitude 25%, pulse 50%); and PEF—pulsed electric field (30 kV cm^−1^, 100 Hz).

**Table 4:** Mutual correlations of hurdle technology parameters on polyphenolic content, antioxidant activities, SSC and pH.

	Storage	HPU Exposure	PEF Exposure	TPC ^1^	HCA ^2^	FL ^3^	CT ^4^	DPPH ^5^	FRAP ^6^	SSC ^7^	pH
Storage	1	0	0	−0.53 *	−0.81 *	−0.55 *	−0.40 *	−0.93 *	−0.24	−0.09	−0.29
HPU exposure		1	0	−0.50 *	−0.31	−0.46 *	−0.49 *	0.02	−0.36 *	−0.23	0.11
PEF exposure			1	−0.30	−0.20	−0.15	−0.25	0.11	−0.13	−0.26	−0.27
TPC				1	0.51 *	0.69 *	0.75 *	0.43 *	0.55 *	0.02	0.05
HCA					1	0.68 *	0.39 *	0.77 *	0.29	0.36 *	0.30
FL						1	0.60 *	0.51 *	0.45 *	0.21	0.08
CT							1	0.37 *	0.74 *	−0.14	−0.06
DPPH								1	0.17	0.15	0.30
FRAP									1	−0.22	−0.22
SSC										1	0.50 *
pH											1

* Correlation is significant at the *p* ≤ 0.05; ^1^ TPC—total phenolic content (mg 100 g^−1^); ^2^ HCA—hydroxycinnamic acid (mg 100 g^−1^); ^3^ FL—flavonol (mg 100 g^−1^); ^4^ CT—condensed tannin (mg 100 g^−1^); ^5^ DPPH assay (µmol 100 g^−1^); ^6^ FRAP assay (µmol 100 g^−1^); and ^7^ SSC—soluble solids content (°Brix). HPU—high-power ultrasound (amplitude 25%, pulse 50%); and PEF—pulsed electric field (30 kV cm^−1^, 100 Hz).

**Table 5:** Optimal hurdle parameters for maximum content of polyphenols and antioxidant activity in the samples.

	TPC	HCA	FL	CT	DPPH	FRAP
Storage (days)	0.0	0.0	0.0	0.0	0.0	0.0
HPU treatment (min)	2.5	4.7	2.5	2.5	5.0	2.5
PEF treatment (min)	1.5	1.5	4.5	1.5	4.5	3.8
Optimal quantity (mg 100 g^−1^)	129.35	39.15	18.04	117.19	294.25	987.75

TPC—total phenolic content (mg 100 g^−1^); HCA—hydroxycinnamic acid (mg 100 g^−1^); FL—flavonol (mg 100 g^−1^); CT—condensed tannin (mg 100 g^−1^); antioxidant activity—DPPH (µmol 100 g^−1^) and FRAP (µmol 100 g^−1^); storage (days); HPU—high-power ultrasound (amplitude 25%, pulse 50%, measured in min); and PEF—pulsed electric field (30 kV cm^−1^, 100 Hz, measured in min).

## Data Availability

The original contributions presented in the study are included in the article, further inquiries can be directed to the corresponding author.
